# Opportunities, challenges, and difficulties in NMR-based metabolomics applied to neovascular age-related macular degeneration (nAMD) patient follow-up

**DOI:** 10.3389/fmolb.2024.1449226

**Published:** 2025-01-28

**Authors:** M. Schoumacher, V. Lambert, M. Campas, P. Blaise, B. Locht, M. Thys, E. Duchateau, E. Cavalier, J.-M. Rakic, A. Noël, P. de Tullio

**Affiliations:** ^1^ Clinical Metabolomics Group (CliMe), Center for Interdisciplinary Research on Medicines, Université de Liège, Liège, Belgium; ^2^ Department of Medical Chemistry, University Hospital of Liège, Liège, Belgium; ^3^ Department of Ophthalmology, University Hospital of Liège, Liège, Belgium; ^4^ Laboratory of Tumor and Development Biology, GIGA, Université de Liège, Liège, Belgium

**Keywords:** age-related macular degeneration, metabolomics, NMR, personalized and precision medicine, biomarkers

## Abstract

**Introduction:**

This study applies NMR-based metabolomics to investigate neovascular age-related macular degeneration (nAMD), addressing challenges in patient management, disease progression evaluation, and treatment response assessment. A two-year follow-up of 29 nAMD patients undergoing treatment provided 231 time points for analysis.

**Methods:**

Over the two-year period, 11 males and 18 females (aged 61–92 years) were monitored, yielding 231 time points. At each time point, blood samples for NMR metabolomics analysis, clinical measurements (e.g., lactate, glucose levels, HDL/LDL cholesterol, and blood pH), and optical coherence tomography (OCT) images were collected to evaluate the progression of choroidal neovascularization. 1H-NMR metabolomic analysis led to the quantification of over 60 metabolites and of the major lipoprotein fractions. Both multivariate and univariate statistical approaches tailored for longitudinal data were employed to identify biomarkers correlating metabolomic changes with ocular alterations during disease progression.

**Results and Discussion:**

Despite a rigorous analytical workflow enabling precise quantification of over 60 metabolites and the application of advanced statistical tools for longitudinal data, achieving consistent results across the cohort proved challenging. The dataset’s heterogeneity, reflecting real-world clinical practice, complicated the derivation of global conclusions. Personalized analyses on a patient-by-patient basis successfully identified individual correlation models, but a universal model remained elusive. This study highlights the inherent challenges of translating findings from controlled settings into clinical practice, where factors such as visit frequency, treatment variability, and disease heterogeneity limit data uniformity. We emphasize the importance of experimental design in longitudinal studies, particularly when dealing with incomplete and variable datasets. We are therefore confident that, considering both the challenges and difficulties identified in this work and the preliminary results presented here, it is possible to develop predictive and individualized models for monitoring patients with nAMD. Such models could greatly assist clinicians in providing better care for these patients.

## 1 Introduction

Age-related macular degeneration (AMD) is the leading cause of vision loss among the aging population in Western countries (Colijn et al., 2017). The prevalence of AMD increases with age, and given the growth of our life expectancy, the number of projected cases is likely to rise in a dramatic manner. In 2014, Wong et al. projected an increment of 47% between 2020 and 2040, reaching 288 million cases (Wong et al., 2014). Clinically, AMD is classified into three stages, and 90% of the vision loss caused by AMD happens in the last stage, named neovascular nAMD (Bressler et al., 2011). nAMD is a degenerative disease. Its onset may be subtle to detect for either patient or physician, and the most advanced stages are characterized by the occurrence of macular neovascularization (MNV), which leads to alternating active bleeding and stationary phases. The earlier nAMD is diagnosed, the later the patient will undergo severe and irreversible visual impairments. For this reason, efforts are being made to raise awareness of regular nAMD screening among people aged 50 and over. Thus, in addition to the general ophthalmologic examination procedures, routine home monitoring between visits is recommended. The diagnostic protocol includes fluorescein angiography (FA) and optical coherence tomography (OCT), and it is generally assumed that better outcomes are achieved with better initial visual acuity (VA) and, therefore, an earlier diagnosis. Unfortunately, lesions are still usually detected when there is already considerable damage causing severe visual impairment. Herein, when nAMD is diagnosed, the only way for the patient to recover VA and stop disease progression is an intravitreal injection of the inhibitor of the angiogenic protein vascular endothelial growth factor (VEGF).

Intravitreal anti-VEGF therapy is the only treatment that allows nAMD patients to recover visual acuity. Indeed, the current therapeutic strategy for managing neovascular AMD is a protocol of a fixed monthly dose of antiangiogenic drug for a 96-week follow-up period (Brown et al., 2006; Rosenfeld et al., 2006). Despite the risk of overtreatment and safety (infection, retinal detachment, development of geographic atrophy (GA), and systemic side effects), monthly dosing guarantees the maximum efficacy in terms of VA benefits (Schmidt-Erfurth et al., 2014; Meyer et al., 2011; Grunwald et al., 2014; Modi et al., 2015). Some studies investigated the possibility of an individualized dosing regimen based on imaging parameters to overcome these safety problems (an “as needed” approach named PRN). The results of these less frequent but more adequate regimens were first encouraging in clinical studies but failed in real-case clinical practice (Lalwani et al., 2009; Holz et al., 2011; Writing Committee for the UK Age-Related Macular Degeneration EMR Users Group, 2014; Chakravarthy et al., 2013). This failure could be related to the fact that OCT-based monitoring is usually applied in a less rigorous manner than in clinical trials. Analysis of OCT images is a fastidious, operator-dependent, and time-consuming process that is not adapted to “real-life” clinical practices. Indeed, the adaptation and personalization of the treatment require a better definition and characterization of the patients’ status to predict the short-term pathology evolution. Then, sensitive and robust biomarkers or predictive models based on OCT and/or biofluid analyses are mandatory in order to allow precise individual management of the disease and help clinicians manage a PRN approach.

As part of the omics sciences, clinical metabolomics is the comprehensive measurement of the metabolites present in a biological human sample. Its main aims are to measure changes in metabolite levels that occur in response to a pathological stress or condition (Clish, 2015; Nicholson et al., 1999) and to provide sets of biomarkers able to assess disease diagnostic, evolution, and/or treatment responses at a personalized level (Ashrafian et al., 2021). Used in combination with cutting-edge big data analysis tools (Blaise et al., 2021; Liland, 2011) to decipher the complex architecture of the generated datasets, this methodology could lead to an innovative representation of an individual’s health status (Clish, 2015). In the context of AMD, several studies were conducted to investigate metabolome changes among diverse biofluids of AMD patients at different stages of the disease (Brown et al., 2018). These approaches identified different compounds belonging to the oxidative stress and energetic pathways, to inflammatory processes, or to lipid metabolism, but none were found to be used in clinics (Osborn et al., 2013; Laíns et al., 2018; Mitchell et al., 2018), and none explored patient treatment and follow-up.

In a previous study, through the use of an untargeted NMR-based metabolomics approach, we reported the functional role of lactate in AMD and highlighted the changes among the lipoprotein profile toward an increase of VLDL moieties both associated with MNV development and progression (Lambert et al., 2020). Based on these results, these two biomarkers could be expected to provide a new tool to monitor the evolution of the pathology and the occurrence of MNV active bleeding phase and pave the way to a new patient follow-up and personalized medicine approach for nAMD treatment (Lambert et al., 2020). Indeed, as these biomarkers are associated with the MNV status of nAMD patients, a predictive model that includes lactate level and lipoprotein profile could be a keystone for an innovative approach to PRN clinical decisions for intravitreal injections of anti-VEGF. As PRN regimens have been shown to lack “handleable” markers of pathology, we were convinced that discovering new MNV biomarkers in nAMD could fill the gap between scientific evidence and clinical practices (Schmidt-Erfurth et al., 2014).

Therefore, we planned to verify the applicability and validity of the previously identified biomarkers in a study closer to clinical reality. For this purpose, we followed 29 patients with established nAMD and under anti-VEGF treatments over 2 years. At each ophthalmologic visit, OCT pictures, VA data, clinicians’ comments, and blood samples were collected. NMR-based metabolomics analyses of the blood samples, as well as measurement of some selected biochemical analyses, were performed. This study seeks to implement an innovative longitudinal approach within a real-world clinical context, aiming to correlate metabolome dynamics, particularly metabolites previously identified as biomarkers of the active phase of nAMD, with longitudinal changes in OCT and/or VA markers used to detect and predict the onset and progression of MNV. By applying this metabolomic approach in a less controlled but clinically relevant setting, we aim to provide clinicians with insights to develop more personalized and effective treatment protocols. This real-world application will help uncover the opportunities, challenges, and limitations of using metabolomics for monitoring patients with AMD.

## 2 Material and methods

### 2.1 Study approval

The human study was conducted under protocols approved by the Ethical Committee of the University Hospital of Liège, B707201523572 (Belgium). Informed consent was obtained from all study subjects before participation.

### 2.2 Patient selection, clinical data, and sample collection

Over a 2-year period, 11 male and 18 female patients, aged 61–92 years (mean age: 76.83 ± 7.6 years, mean BMI 26 ± 3), were followed in this study. All participants, free from metabolic diseases such as diabetes (self-reported and no medical record found) and no severe obesity, were diagnosed with exudative AMD and enrolled in a monthly intravitreal anti-VEGF injection regimen (ranibizumab or aflibercept). At each visit, OCT retinal images were collected, and visual acuity (VA) assessments were performed by practitioners. The VA outcomes and OCT analyses were used by the practitioners to adjust the treatment regimen and schedule subsequent visits. No special interventions were applied for this study, as we aimed to replicate routine patient care as closely as possible. At each ophthalmologic visit, blood samples (plasma EDTA for metabolomics analysis, serum for lactate, glucose, and HDL/LDL cholesterol, and whole blood for pH determination) and OCT images were collected (total number of visits = 231). For each visit, lactate concentration, blood pH, and HDL/LDL cholesterol were measured. Lactate, glucose levels, and HDL/LDL cholesterol values were obtained using dedicated enzymatic dosage kits from Alinity® (lactic acid, ultra HDL, direct LDL, and glucose reagent kits). Blood pH was measured using a GEM 500 Premier analyzer (Werfen, Barcelona, Spain). Collected plasma samples were conserved at −80°C prior to sample preparation and proton NMR metabolomics analysis.

### 2.3 OCT images analysis

**FIGURE 1:**
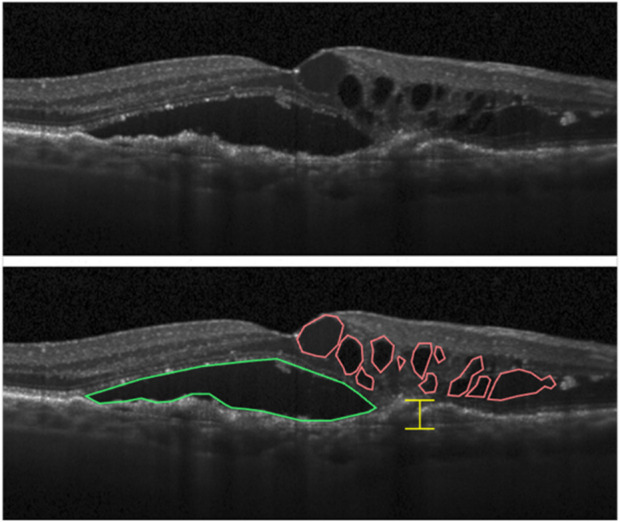
Markers of nAMD that can be highlighted from OCT images of retinas. Intraretinal cystoid fluid (IRC) accumulation is highlighted in red, subretinal fluid (SRF) in green, and measurement of the largest pigment epithelial detachment (PED) in yellow (Schmidt-Erfurth and Waldstein, 2016).

Analysis of OCT images (Figure 1) is crucially important because it gives access to the best visualization of the pathological event occurring during the follow-up of patients and to the pathological status of the participants. All data were acquired on a Heidelberg HRA + OCT (Heidelberg Engineering Gmbh, Heidelberg, Germany) by the Experimental Ophtalmology Laboratory of Liège team. Images were analyzed by our team following guidelines described in the literature to assess AMD status (Schmidt-Erfurth et al., 2014; Schmidt-Erfurth and Waldstein, 2016). Three main markers were measured and followed: intra-retinal cystoid fluids (IRC), pigment epithelium detachment (PED), and subretinal fluids (SRF). IRC and PED measurements are used to monitor nAMD progression. Indeed, the presence of IRC fluids is linked to MNV processes that are the hallmark of nAMD, and PED growth is reported to be associated with long-term vision loss in a flexible treatment regimen (Schmidt-Erfurth and Waldstein, 2016). On the other hand, the presence of SRF fluids is a sign of healing retinas and is the only measured parameter that is correlated with improved visual function (Schmidt-Erfurth and Waldstein, 2016).

At each ophthalmologic visit, 12 scans were taken from each eye. From each scan, the surface of IRC spots (in mm^2^), SRF spots (in mm^2^), and the largest PED section were evaluated from the initial pigment epithelium baseline (in μm). Measurement was performed using Heidelberg Eye Explorer software. From the 231 visits, a total of 5,544 images (12 scans/eye/visit) were generated, and 16,632 measures (three measures/scan/eye/visit) were obtained, providing a single value for each parameter at each single time point.

### 2.4 NMR metabolomic analysis

^1^H-NMR metabolomic and lipoprotein profiles were obtained from plasma samples collected during the 2 years of follow-up and conserved at −80°C. For the lipoprotein profile, determination samples were measured at 298K on a Bruker Neo spectrometer (Bruker, Billerica, United States) operating at 500.13 MHz for proton detection. The NMR instrument is equipped with a TCI 5 mm cryoprobe equipped with *Z* gradients. Maleic acid was added to samples as an internal standard, allowing quantification, and trimethylsilyl-3-propanoic acid-*d*_4_ sodium salt (TMSP) was used for ppm calibration. For sample preparation, 500 μL of plasma was mixed with 200 μL of deuterated phosphate buffer, to which was added 100 μL of 35 mM maleic acid solution and 30 μL of 10 mg/mL TMSP solution in D_2_O. The final solution was vortexed and placed in a new 5 mm NMR tube before being analyzed. For the NMR analysis, an edited 1D-CPMG sequence with water pre-saturation was used: RD-90-(-t-180-t)n with a relaxation delay of 4 s (RD), a spin echo delay of 300 ms (t), and a 128 loop (n). The pulse for water pre-saturation occurred during the relaxation time (RD), and the number of scans was fixed at 64. The total acquisition time is 3.1981568 s with four dummy scans.

For metabolite quantification, all NMR samples prepared as described above and already analyzed for lipoprotein profile evaluation were filtered through a wash-up 10K filter (AMICON ultra 0.5 mL-10 KDa filter tube, Merck-Millipore, Burlington, Massachusetts, United States) for 60 min at 13,000 rpm at 4°C. A 270 μL aliquot of filtrate was added to 30 μL of calcium formate 5 mM solution and placed in 3 mm NMR tubes. All samples were measured at 298K on a 700 MHz Bruker Avance HD spectrometer operating at 700.17 MHz for proton detection. The sequence used is a 1D NOESY sequence with pre-saturation for urine samples. The NOESY-presat experiment used an RD-90°-T1-90°-Tm-90°-acquire sequence with a relaxation delay of 4 s, a mixing time (Tm) of 10 ms, and a fixed T1 delay of 4 μs. A water suppression pulse was placed during the relaxation delay (RD). The number of transients was 64 (64K data points), and four dummy scans were chosen. The acquisition time was fixed to 3.2769001 s. Internal standards and deuterated buffers were purchased from Sigma-Aldrich (St. Louis, Missouri, United States).

### 2.5 Lipoprotein profile evaluation

The NMR lipoprotein profile was evaluated from data recovered using 1D-CPMG NMR analysis of plasma samples using the methods previously described (Lambert et al., 2020). Then, we determined the chemical shift corresponding to four lipoprotein fractions: F1 = 0.92 ppm (mainly VLDL), F2 = 0.91 ppm (mainly IDL and LDL), F3 = 0.89 ppm (mainly LDL and IDL), and F4 = 0.88 ppm (mainly HDL). The signal intensity at these different chemical shifts was measured and then normalized to the total intensities of all fractions to reduce the impact of the global lipoprotein concentrations that could differ between samples. Thus, the obtained values represent a fraction of the total signal and allow a comparison of the different lipoprotein profiles across all plasma samples.

### 2.6 ChenomX^®^ metabolite quantification

Metabolite concentration was measured by ^1^H-NMR using spectral data from the analysis of filtered plasma samples. Spectral deconvolution was achieved using the ChenomX® NMR v9 suite software (Chenomx Inc., Edmonton, AB, Canada) in profiler mode by manually fitting the resonance peak of 61 metabolites. The quantification was based on the signal of the chosen reference, calcium formate at 8.46 ppm (a list of all fitted metabolites and chemical shift cluster is available in Supplementary Table S1). As we wanted to measure the lipoprotein profile on the sample, it was not possible to follow the ChenomX® SOP guidelines. Thus, the concentration values obtained are not absolute but rather relative. This step allowed us to analyze the variations among the different metabolite concentrations among all measured samples.

### 2.7 Statistical analysis

All analyses were made using dedicated R packages, and raw data are accessible in the following GitHub repository: https://github.com/MS28uliege/nAMD-FiBMS-data.git.

For unsupervised principal component analysis (PCA), the *MixOmics* R package was used to generate the model and the score and loading plots representing the variation within the dataset. Data were centered and scaled to unit variance for model generation. For the model generated from the entire dataset (with all patients combined), multilevel normalization was applied to account for the longitudinal nature of the data. This method is based on the “split-up” variation approach developed by Westerhuis et al. (2010) and allows extracting the stimulation effect from each subject by removing the between-subject effect. Hence, we get a better representation of the change effects within the subject on the score plot than by considering all sources of variation.

To investigate correlations between OCT markers and quantified metabolites, we applied PLS2 regression using the *MixOmics* R package. Model performance, using the *perf()* function, for each PLS regression was assessed through 10-fold cross-validation, repeated five times, to evaluate the *R* ^2^ and overall Q^2^ of the model. In cases where the sample size was too small for 10-fold cross-validation (n < 10), such as for PS32, we used 5-fold cross-validation instead. During this analysis, correlations with absolute values exceeding 0.5 were considered noteworthy, and those greater than 0.7 were considered strong.

Other correlation analyses and plots were generated using the *cor()* function in R, with correlations typically considered interesting if their absolute value exceeded 0.5 and considered strong if above 0.7.

## 3 Results

**FIGURE 2:**
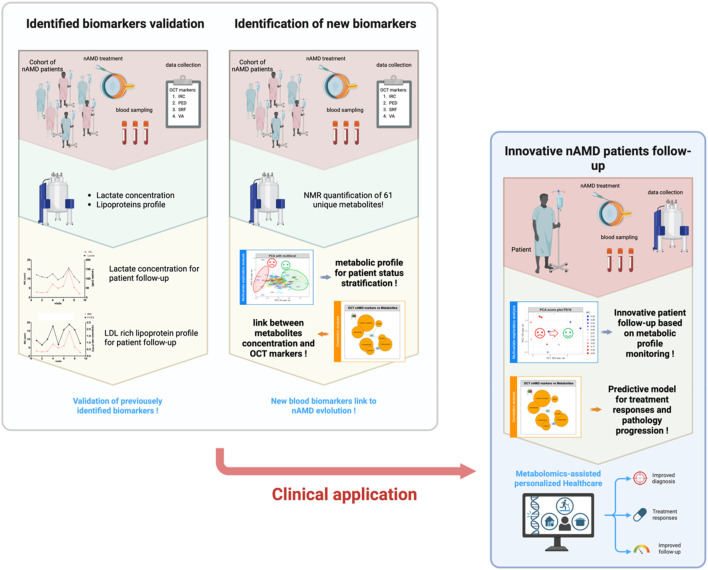
Overview of the study strategy and workflow, progressing from the validation of previously identified biomarkers for individual monitoring and cohort analysis to the identification of new blood biomarkers associated with patient status. This approach aims to establish an innovative healthcare model for nAMD patients, leveraging rational monitoring of blood marker levels to track disease progression and predict treatment responses.

Our cohort comprises 29 selected patients, monitored over 231 time points, with an average of 7.9 visits per patient across 2 years of treatment. Three datasets were generated from this cohort (see Figure 2). The first consists of ophthalmologic data (IRC, SRF, PED, and VA: means and range for each patient; see Supplementary Table S3). The second includes the quantification data of previously identified biomarkers, namely, lactate levels and lipoprotein profiles (fraction F1/VLDL to F4/HDL), but also blood pH and HDL/LDL cholesterol ratio (the means and range for each patient are in Supplementary Table S4). The third combines NMR-measured metabolome data from metabolomics with biochemical data and gives us an overview of the global metabolome (a table of mean relatives concentration values and standard deviations for each metabolite is provided in supplemental info Supplementary Table S2; mean metabolite concentrations and standard deviations for each individual are available in the following GitHub repository: https://github.com/MS28uliege/nAMD-FiBMS-data.git). In this study, having relative quantification values is not a limitation because we are focused on variations in metabolite concentrations in relation to disease progression.

As shown in Figure 2, our data analysis and utilization strategy were sequential and guided by the results of each analytical step, beginning with a comprehensive approach that utilized all available data to develop a more personalized strategy.

To align with the project’s primary goal, validating previously identified biomarkers, our initial approach focused on using these molecules, specifically lactate levels and lipoprotein profiles, to examine correlations between their variations and changes in AMD OCT markers. These markers (IRC, PED, SRF, and VA) offer insights into the patient’s condition and disease progression. Establishing a correlation between changes in lactate levels and lipoprotein profiles with IRC and PED would validate our previous findings and could pave the way for a novel follow-up strategy. This strategy would utilize these markers to assess patient status and predict treatment responses.

To gain a more comprehensive understanding of the metabolic changes occurring during the progression and treatment of the disease, we also aimed to track the evolution of patients’ metabolomic profiles using our NMR-based metabolomics datasets. With innovative data visualization tools, we sought to identify patient clusters through unsupervised statistical analysis, grouping patients who exhibit similar disease progression patterns. This approach would help classify patients and identify those who may not respond to intravitreal anti-VEGF injections.

Additionally, by leveraging the extensive data generated from both metabolite quantification and OCT biomarker analysis, we aimed to explore the potential relationships between metabolite levels and OCT marker values. If such links could be established, they might enable the identification of new biomarkers capable of explaining patients’ MNV status. Together with the previously identified biomarkers, this would allow us to build a more robust and comprehensive tool for evaluating patient status, predicting treatment responses, and gaining further insights into the complex pathological processes underlying the progression and treatment of this degenerative disease.

Finally, driven by the results obtained with our previous approaches, we plan to evaluate our methodology at an individual level, exploring whether applying this strategy on a personalized basis could enhance the understanding of disease evolution. As patients may progress differently, an individualized approach could prove more effective or, at the very least, validate our findings when investigating the relationship between metabolomic and OCT marker data.

### 3.1 Previously identified biomarkers poorly correlated with OCT markers of nAMD

Correlation analysis investigating potential relationships between OCT/VA disease markers and previously identified biomarkers (lactate and lipoprotein profile) revealed only weak associations between the two datasets (Supplementary Figure S1A). Additionally, progression patterns for both disease markers and biomarker values varied widely among individuals (Supplementary Figures S1B–E), with no discernible trends emerging from these parameters.

### 3.2 Untargeted NMR-base metabolomics failed to identify an informative cluster of patients

The multilevel normalization provided a good representation of the individual evolution, as no strong cluster composed of samples issued from the same individual can be observed (Supplementary Figures S2A, C). Nevertheless, the generated PCA models highlighted poor variability among our datasets (expl. var: PC1 = 18%, PC2 = 8%, PC3 = 5%, and PC4 = 5%). Indeed, the PCA score and loading plot were poorly informative as no group of patients seems to exhibit a distinct metabolic profile from the remaining cohort (Supplementary Figures S2C, E). Moreover, as depicted by the loading plot of PC1 to 4 and their explained variance (Supplementary Figures S2D, F), the variations of metabolites among the dataset were quite weak. It is important to note that PCA led us to identify two outliers that were removed from the dataset used for the following statistical analysis. These two outliers account for two samples from the same patient and presented abnormal overall levels of metabolite concentrations (Supplementary Figure S3). No outliers are associated with the highest BMI score.

### 3.3 Metabolite levels show a weak association with OCT markers when analyzing all patients collectively

Pearson correlation tests were performed to analyze linear association or dependency between NMR metabolite concentrations and ophthalmologic data (OCT markers and VA). From this analysis, none of the metabolomics variables were interestingly correlated with the OCT or visual acuity data, indicating weak linear associations between those parameters (Supplementary Figure S4).

**FIGURE 3:**
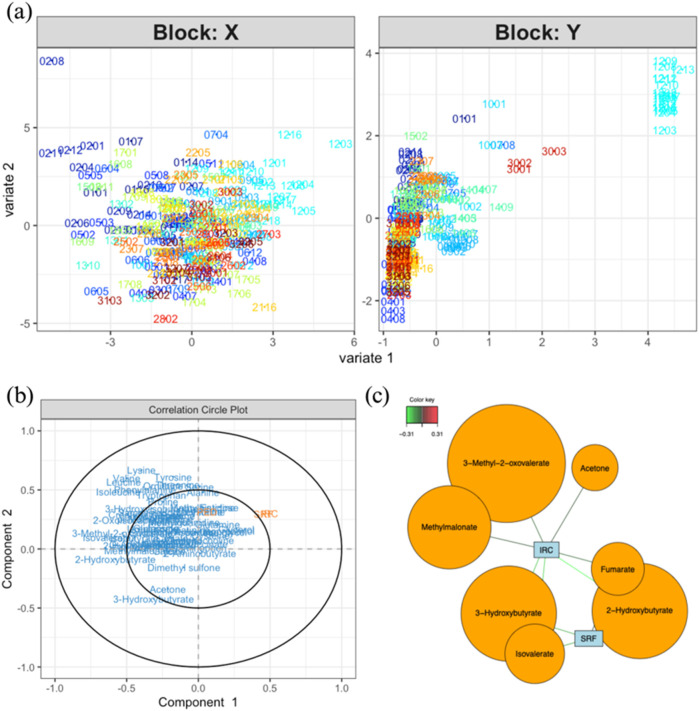
**(A)** Score plot for the PLS analysis (model performance is assessed using R^2^ values for each Y and overall Q^2^ values; component 1: IRC. R^2^ = 0.167, PED. R^2^ = 0.005, SRF. R^2^ = 0.14, totalQ^2^ = 0.089; component 2: IRC. R^2^ = 0.005, PED. R^2^ = 0.007, SRF. R^2^ = 0.001, totalQ^2^ = −0.039) of data coming from the measured metabolites (X) and OCT data (Y). **(B)** Correlation circle plot showing the correlations between variables and components (explain how to use). **(C)** Relevance network representing the interesting correlations between metabolites and OCT measurements. The color of the lines indicates the strength of the correlation (correlations with an absolute value smaller than 0.5 are not worth considering). *Notes: bubble dimensions are a function of the name variables and have no statistical significance*. In these score plots, the sample code XXYY represents the individual number and the visit number (e.g., 0101 for the first visit of patient 01).

To consider the multivariate aspect of the dataset, partial least square regression 2 (PLS2) was performed to highlight putative interesting metabolites able to describe variations among OCT data. From the PLS model, the score plot (Figure 3A) represents all samples regarding the measured NMR metabolites concentrations (X) and measured parameters in OCT images (Y). The corresponding correlation circle plots (Figure 3B) show the variables correlated with the different components. Some variables appear weakly correlated with IRC and SRF values and could be worth considering (i.e., 3-methyl-2-oxovalerate, methylmalonate, acetone, 2-hydroxybutyrate, fumarate, isovalerate, and 3-hydroxybutyrate). A relevance network plot (Figure 3C) can be generated in which the correlations are represented. In this plot, we can identify some features that are correlated to IRC and SRF values. It is important to note that the tuning threshold for the relevant association network had to be lowered to generate the network. Indeed, no association stronger than 0.31 was found. From this analysis, some interesting features can be highlighted, such as metabolites correlated with important OCT markers for AMD evolution. However, these results should be interpreted with caution, as the cutoff for variable selection in the PLS analysis was lowered, suggesting that the findings may lack robustness.

### 3.4 Individual follow-up of AMD patients

**FIGURE 4:**
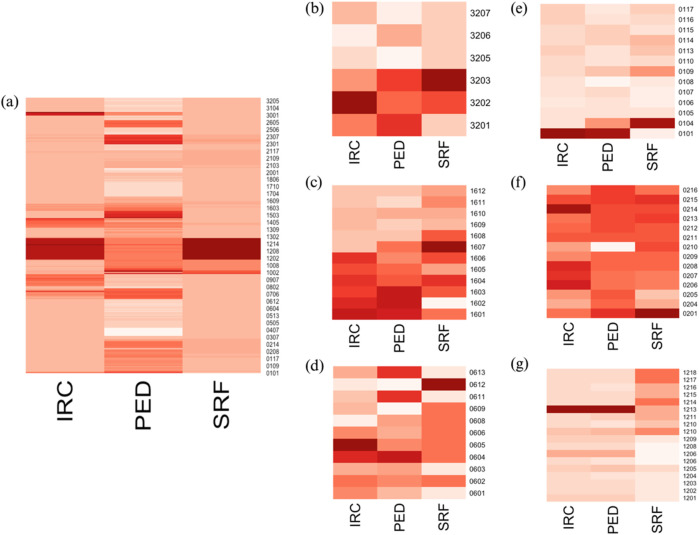
**(A)** Heatmap of OCT data for all patients; heatmaps of OCT data for individual patients: **(B)** PS16, **(C)** PS32, **(D)** PS06, **(E)** PS01, **(F)** PS02, and **(G)** PS12. The *x*-axis represents each visit, while the *y*-axis shows the measured OCT values. In the right panels **(B–G)**, it is evident that patients evolved at different rates. Some patients appear to show positive improvements in their status during follow-up **(B–D)**, while others exhibit minimal changes over the study period **(E–G)**. These distinct progression patterns are apparent when examining patients individually but are not reflected in the left panel **(A)**, where individuals with high OCT marker values dominate, masking the smaller changes in those with lower values.

As seen in the previous analysis, the significant heterogeneity within the cohort regarding patients’ disease progression may hinder the development of robust and global statistical models to describe and predict nAMD evolution. When comparing changes in OCT values across selected patients, it is clear that each patient exhibited a unique pattern of disease progression. Some patients experienced notable changes during their follow-up, while others remained quite stable throughout the study. The variability in patient progression complicates the identification of comparable individuals in terms of disease status, injection frequency, visit frequency, and the severity of the pathology at enrollment (all summarized in Supplementary Table S3). To more effectively capture the metabolic variability of patients during treatment and follow-up and to link this variability with their pathological conditions, we aimed to conduct individual analyses for each patient. When generating the heatmap of OCT values for all patients, it is evident that some patients exhibit higher values than others, indicating that they are progressing from different baseline conditions. Moreover, the recovery, stabilization, or deterioration of ocular health varies among patients, with each experiencing different levels of progression (Figure 4). Analysis of these heatmaps highlighted the heterogeneity in patient status and progression. Some patients showed negative changes during their follow-up (Figures 4B–D), while others remained relatively stable, displaying neither significant improvement nor deterioration throughout the study period (Figures 4E–G). In general, it appears that each patient follows their own trajectory in terms of care and disease progression, with few changes in visual status from one visit to the next.

This heterogeneity in disease progression and treatment responses led us to consider a more individualized approach to the data. To explore whether our approach can yield meaningful results at the individual level, we applied the statistical workflow independently for each patient. Specifically, PCA analyses were conducted to represent the metabolic evolution of each patient throughout the follow-up period and highlight visits that exhibited similar outcomes. This approach might allow us to build a statistical model capable of representing patient status and analyzing their trajectories. Additionally, PSL2 regression analyses were employed to investigate the relationship between variations in the metabolome of each patient and the evolution of data obtained from the analysis of recorded OCT images (IRC, PED, and SRF). Several relevant examples are presented below. We showcase the results from two categories of patients in this paper: those who exhibited changes in status during their follow-up (PS16, PS32, and PS06) and those who did not show notable changes in their OCT markers throughout the study (PS01, PS02, and PS12). These examples were selected because they provide a clear and visual representation of the phenomena discussed in this section. Indeed, the classification is quite subjective for the other members of the cohort, and results are more confusing and more difficult to interpret, as discussed below. A summary of relevant results from all patients is provided in Supplementary Tables S5, S6. Supplementary Table S5 includes the performance test results for all PLS models generated for each patient, while Supplementary Table S6 lists the identified metabolites that show a strong correlation (|cor| ≥ 0.7) with at least one of the measured OCT parameters. Specific examples are discussed below, followed by a general overview of the entire cohort.

**FIGURE 5:**
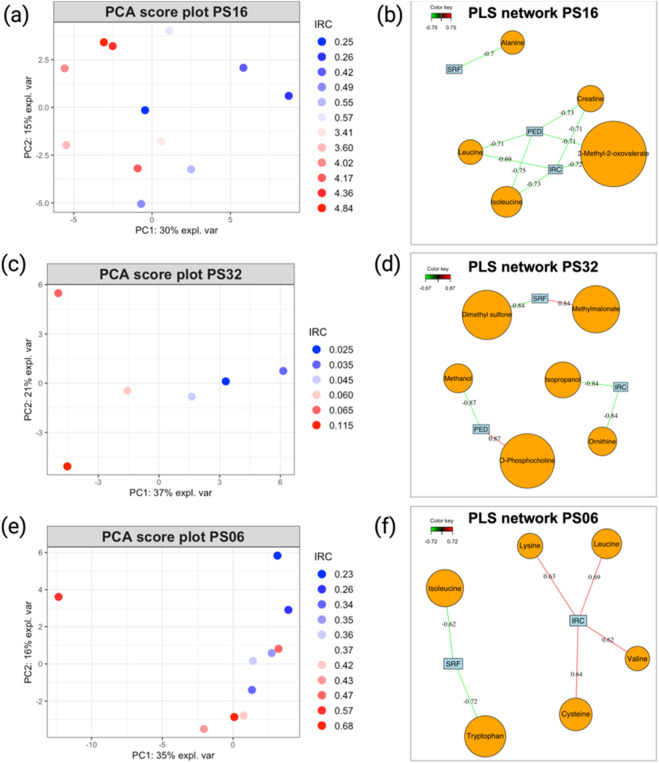
**(A)** PCA score plot illustrating the evolution of patients PS16, **(C)** PS32, and **(E)** PS06. Sample colors correspond to the IRC values from each visit, with higher values represented in red and smaller values in blue. **(B)** PLS2 variable correlation network, showing the relationship between metabolite variables and OCT data for patients PS16, **(D)** PS32, and **(F)** PS06. Only correlations with an absolute value ≥0.7 are considered strong. Model performance is evaluated by overall Q^2^ values, as detailed in Table 1, and metabolites, as shown in Table 2.

The PCA analysis performed on the NMR metabolite profiles of patients PS16, PS32, and PS06 revealed interesting results as the metabolomics data evolved in line with the patients’ ophthalmological progression. In fact, by examining each score plot (Figure 5A for PS16, 5c for PS32, and 5d for PS06), we can observe that samples with higher IRC values, probably the most representative marker of the disease evolution, tend to form distinct clusters compared to those from visits associated with lower values. This data representation provides a clear way to identify periods of positive progression in patients and may be useful for analyzing their overall evolutionary trend (for loading plots of PCA models, please refer to Supplementary Figure S5).

For example, a closer look at the PCA score plot for PS16 reveals that all samples with elevated IRC values are positioned on the left side of PC1, which accounts for 30% of the total variance. This suggests that these events have a significant impact on the patient’s metabolism. Moreover, the PLS2 models generated showed satisfactory performance (Table 1), and the PLS correlation network ((Figure 5B for PS16, 5d for PS32, and 5e for PS06) offered a clear visualization of the metabolites strongly correlated with the disease markers (IRC, PED, and SRF). Several metabolites with strong correlations were identified (Table 2).

**TABLE 1:** This table shows the PLS2 prediction Q2 values from all models discussed above.

Feature	Patients presenting nAMD status evolution			Patients without clear nAMD status evolution		
	PS16 total.Q2	PS32 total.Q2	PS06 total.Q2	PS01 total.Q2	PS02 total.Q2	PS12 total.Q2
IRC	0.4397830	0.1170016	0.1241289	−0.4144425	−0.3759959	−0.3334529
PED	0.3400849	0.5389380	0.1880756	−0.3570862	−0.2153993	−0.3319730
SRF	−0.3214319	−0.3976716	−1.1675381	−1.2896416	−1.3308868	0.5713058
IRC range	0.251–4.845	0.025–0.115	0.235–0.680	0.045–8.411	0.000–0.180	20.040–22.385
PED range	360.485–535.333	245.667–273.167	231.750–274.250	409.333–665.667	280.917–540.333	497.750–595.917
SRF range	0.000–0.516	0.005–0.030	0.000–0.010	0.000–0.330	0.315–1.025	20.050–20.080

Positive Q2 values close to 0.5 can be considered a predictive model, while negative Q2 values indicate models that have very poor predictability capacities and are not worth considering.

**TABLE 2:** This table shows the top five metabolites extracted from the PLS2 correlation network representation discussed above.

Patient Id	Top 5 PLS metabolites (corr values/Y)
PS16	3-Methyl-2-oxovalerate (−0.72/IRC; −0.71/PED) - creatine (−0.71/IRC; −0.73/PED) - isoleucine (−0.73/IRC; −0.75/PED) - leucine (−0.69/IRC; −0.71/PED) - alanine (−0.7/SRF)
PS32	Isopropanol (−0.84/IRC) - ornithine (−0.84/IRC) - O-phosphocholine (0.87/PED) - methanol (−0.87/PED) - methylmalonate (0.84/SRF)
PS06	Leucine (0.69/IRC) - lysine (0.63/IRC) - cysteine (0.64/IRC) - isoleucine (−0.62/SRF) - tryptophan (−0.72/SRF)
PS02	Succinate (0.48/IRC; 0.5/SRF) - choline (−0.69/PED) - proline (−0.48/PED) - sarcosine (0.47/SRF) - tyrosine (0.58/SRF)
PS12	MyoInositol (0.72/IRC; 0.72/PED) - acetone (−0.80/SRF) - 2-oxoisocaproate (−0.82/SRF) - 3-methyl-2-oxovalerate (−0.73/SRF) - methionine (−0.72/SRF)
PS01	Acetone (0.67/PED; 0.60/SRF) - ornithine (0.57/PED; 0.62/SRF) - 3-hydroxybutyrate (0.72/PED) - 3-methyl-2-oxovalerate (−0.60/PED) - propylene glycol (0.57/PED)

Metabolites that exhibit absolute value |cor| ≥ 0.7 can be considered strongly associated with the effect by the models, while values |cor| < 0.5 are considered weak. The sign of the correlation value indicates whether the correlation is positive or negative. A positive correlation indicates that concentration values increase in concordance with the measured OCT parameter. Note that strong correlations do not account for the predictive ability of the selected features. Only features extracted from validated models (models with Q^2^ ≥ 0.5 from Table 1) can be considered relevant.

**FIGURE 6:**
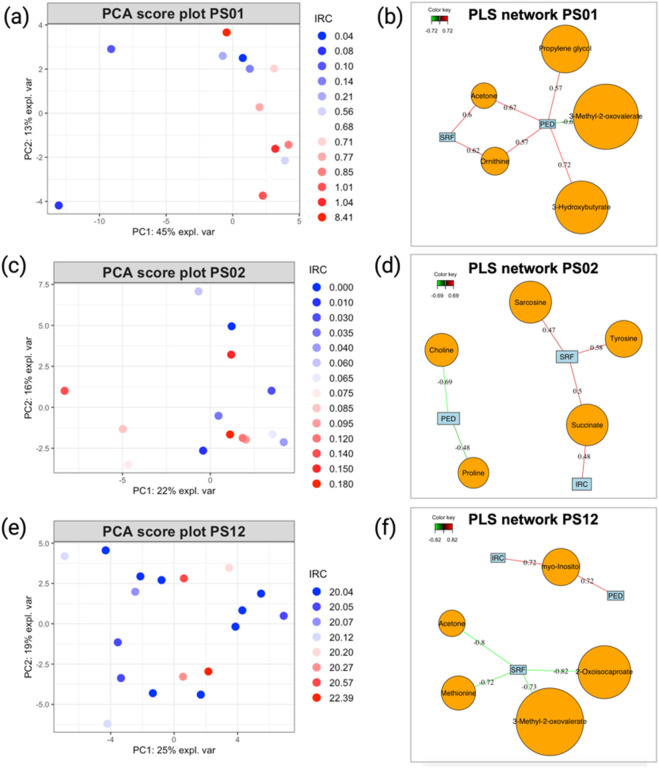
**(A)** PCA score plot illustrating the evolution of patients PS06, **(C)** PS02, and **(E)** PS12. Sample colors correspond to the IRC values from each visit, with higher values in red and smaller values in blue. **(B)** PLS2 variable correlation network, showing the relationship between metabolite variables and OCT data for patients PS01, **(D)** PS02, and **(F)** PS12. Only correlations with an absolute value ≥0.7 are considered strong. Model performance is evaluated by overall Q^2^ values, as detailed in Table 1, and metabolites, as shown in Table 2.

Although the analysis of the previous group of patients was relatively insightful, applying our analytical strategy to patients who exhibited minimal changes during the follow-up proved less informative. In the PCA score plots (Figures 6A,C,D) and the loading plot (Supplementary Figure S6) generated from the metabolomics data of these patients, no clear distinction could be made between samples with positive or negative outcomes based on the IRC parameter, which is the best indicator of disease progression. This observation can also be made when trying to represent the evolution of patients based on other OCT parameters (data not shown). Even when patients experienced extraordinary events (such as extreme values during a single visit, as seen for patients PS01 and PS12), these time points did not appear markedly different from other visits associated with stable outcomes. Similarly, the PLS2 models produced for these patients showed globally weaker correlations (Figures 6B,D,E) than those observed in patients who experienced more significant changes during the follow-up (Figures 5B,D,E). Moreover, PLS2 models exhibited poor performance indicators, showing that correlation values that might appear as strong are not relevant (Table 1). These findings support our hypothesis that, when considering the entire dataset, patients with minimal progression reduce the validity of the model, hindering the identification of meaningful correlations between nAMD OCT markers (IRC, PED, and SRF) and quantified blood metabolites.

The statistical workflow described above was applied individually to each patient within the entire AMD cohort. Supplementary Tables S5, S6 summarize the overall Q^2^ PLS2 values and list the metabolites with strong correlations (|cor| ≥ 0.7) for each model across patients. To illustrate general trends, Tables 1 and 2 display the performance of the PLS2 models and the top five metabolites most strongly correlated with OCT data for selected individuals. Notably, some models demonstrated promising results; for instance, the PLS2 model for patient PS16 achieved a Q^2^ value of 0.44, with metabolites like 3-methyl-2-oxovalerate, creatine, and isoleucine showing correlations above 0.7.

Patients who experienced progression in their nAMD generally exhibited stronger PLS model performance than those whose condition remained stable, as most Q^2^ values in stable cases were negative. However, even within the subgroup of patients with disease progression, the results were inconsistent, with considerable variation in model performance and the specific metabolites identified (Supplementary Table S5). This inconsistency extends across the broader cohort, where no metabolite was consistently linked to AMD progression in all analyses (Supplementary Table S6).

For patients displaying typical AMD progression during follow-up (PS16, PS32, and PS06), the metabolites most strongly associated with disease progression differed among individuals, with each model highlighting distinct key metabolites despite encouraging performance indicators. Conversely, for patients with stable AMD progression throughout the study (PS01, PS02, and PS12), the PLS2 models showed limited predictive performance, making it challenging to draw robust conclusions about the relationship between metabolite level variations and changes in OCT-based AMD markers.

## 4 Discussion

Our previous study, which identified blood lactate level and lipoprotein profiles as putative biomarkers of the active phase of nAMD, prompted us to use these metabolites to predict disease status in a personalized patient follow-up. With this aim, we designed a longitudinal study that was as close as possible to the clinical routine care of advanced and exudative AMD patients under anti-VEGF treatments. The initial plan was to follow volunteer patients for 2 years during their ophthalmological care without any adjustments to their treatment plans or the intervals between visits. Using this longitudinal approach, we hoped to identify transitions between active and stationary phases of the disease at an early stage and link them to metabolomic markers. Initially, we attempted to use the previously identified biomarkers, blood lactate levels, and lipoprotein profiles to represent patient evolution. However, this approach failed to effectively capture patient progression. When we developed and applied a statistical workflow to the entire cohort to identify patient clusters with similar disease progression or to study potential relationships between metabolite concentration values and OCT imaging biomarkers, few significant results emerged. This analysis demonstrated that the substantial heterogeneity within the cohort concerning disease progression likely impedes the development of robust and generalizable statistical models to describe and predict nAMD evolution. For this reason, we shifted to applying our statistical workflow at the individual level. Although some encouraging results were observed for certain patients, the lack of pathological variation in a significant portion of our dataset limits the development of reliable statistical models. The small number of patients showing notable results is insufficient for drawing meaningful conclusions about the entire cohort. This issue is further exacerbated by variability in patient follow-up schedules, with considerable differences in visit timing and, consequently, the number of recorded time points and events for each patient (Supplementary Figure S6).

The complexity of clinical data in the treatment of neovascular age-related macular degeneration (nAMD) is well-documented in the literature. Indeed, the variability in clinical practices, the intricacies of the pathology, and the heterogeneity of patient populations contribute to the generation of heterogeneous and complex data sets (Daien et al., 2021; Polat et al., 2017). For instance, studies have shown that real-world treatment regimens often differ significantly from the rigorous protocols established in clinical trials, leading to varied outcomes in terms of visit frequency and the intervals between them (Bulirsch et al., 2022; Lotery et al., 2017). Clinicians often modify visit schedules based on each patient’s progression of nAMD, which complicates efforts to standardize care (Polat et al., 2017).

Unfortunately, our study, which reflects real-world clinical conditions, has resulted in heterogeneous and complex data sets due to the variability of clinical practices, the complexity of the pathology, and the diversity of our patient group. For example, in terms of the longitudinal aspect, we ultimately obtained a relatively inconsistent matrix of visit schedules and intervals. This timing was determined by clinicians based on the progression of each patient’s nAMD, making it difficult to control and predict. Consequently, and quite logically, patients who responded well to treatment had fewer ophthalmological visits than patients who did not or poorly respond to treatment. This obviously affected the application of statistical models dedicated to longitudinal approaches. When considering the evolution of the pathology, several observations could be made. At the time of entry into the study, all patients were at varying stages of the disease, with notably different degrees of ocular damage. Furthermore, over the 2-year follow-up period, we observed minimal changes in their ocular parameters and disease progression. Therefore, over the entire cohort, we encountered only a few events leading to a change in nAMD and patient status. This is undoubtedly due to the very slow progression of the pathology, the success of anti-VEGF therapy administered to patients, or a combination of both factors. These limitations made it especially challenging to distinguish between active and stationary phases of the disease and to identify transitions between them. This unpredictability led to complex data structures, where changes in disease status were often subtle and difficult to anticipate. Although tracking the dynamics of disease progression is both highly interesting and important, it encounters challenges due to clinical practice constraints and the unpredictability of changes in patient status. Unfortunately, the absence of a control group and the heterogeneity of patients further complicate our approach.

The role of lactate and lipoproteins in the management of nAMD is increasingly recognized in the literature. In our recent work, we demonstrated that lactate, a byproduct of anaerobic metabolism, has been proven to be implicated in the formation of macular neovascularization (MNV), a hallmark of nAMD (Lambert et al., 2020). Furthermore, recent studies have shown that lipid metabolism, particularly involving lipoproteins, plays a crucial role in the pathogenesis of nAMD. For instance, Zhang et al. identified differentially regulated apolipoproteins and lipid profiles as potential biomarkers for both polypoidal choroidal vasculopathy and nAMD, indicating that dysregulation in lipid metabolism may contribute to disease progression (Zhang et al., 2022). Moreover, Cheung et al. demonstrated an association between plasma lipoprotein subfraction concentrations and lipid metabolism in relation to age-related macular degeneration, suggesting that variations in lipoprotein profiles may influence the risk and severity of nAMD (Cheung et al., 2017).

Despite these data and our previous findings, both in a murine model and in a human study, no overall correlation was identified between lactate levels (and lipoprotein profiles) and parameters related to the patient’s ophthalmological condition across all cases and visits. Furthermore, when we analyzed our datasets using an appropriate approach to account for their longitudinal aspect and to correlate metabolite variations with changes in OCT data related to AMD status, no consistent results were found, despite the statistical tests conducted, whether considering all samples together or individualizing the statistical approach. Thus, while the individual analysis of single patients led to interesting information, these results lack coherence when trying to develop a more global model. Interestingly, we were able to identify metabolites common to all cases or present in several patients (i.e., 3-methyl-2-oxovalerate, methylmalonate, acetone, 2-hydroxybutyrate, fumarate, isovalerate, 3-hydroxybutyrate), which may be correlated with the ophthalmological data describing the pathology. Although these data are not statistically significant, they suggest intriguing avenues for further exploration. Indeed, these metabolites have been implicated in the processes of para-inflammation and inflammation, which are critical in the development of neovascularization (MNV) associated with neovascular nAMD. For example, 3-methyl-2-oxovalerate, a branched-chain keto acid, has been shown to be significantly elevated in conditions such as impaired fasting glucose and type 2 diabetes mellitus, which are risk factors for nAMD (Spanou et al., 2022; Menni et al., 2013). Its accumulation is linked to metabolic dysregulation, which can exacerbate inflammatory responses in tissues, potentially contributing to the pathogenesis of MNV (Sampey et al., 2012). Acetone and 2-hydroxybutyrate are also noteworthy in the context of inflammation. Acetone, a ketone body, can influence inflammatory pathways, while 2-hydroxybutyrate has been recognized for its potential anti-inflammatory properties, possibly counteracting some of the pro-inflammatory effects of other metabolites (Wang et al., 2020). The balance between these metabolites may thus be crucial in modulating the inflammatory response relevant to MNV.

In addition, the generated individual models are particularly interesting and represent probably the most relevant point of this study. Indeed, our analyses show that it is possible to generate an individual correlation model for each patient between the ophthalmological data of nAMD and some metabolic biomarkers. Inter-individual variations due to patient genetic background, age, response to treatment and lifestyle, and the heterogeneity of the pathology and its evolution could mask the intra-individual variations linked to nAMD when searching for a global model. For instance, the BIOIMAGE study investigated genetic variants associated with treatment response to aflibercept, emphasizing the significance of these biomarkers in patient stratification (Burés Jelstrup et al., 2020). Additionally, research has shown that specific polymorphisms, such as Y402H in the CFH gene, are linked to varied responses to anti-VEGF treatments, highlighting the necessity of a personalized approach (Hong et al., 2016; Mohamad et al., 2018). These findings indicate that genetic factors should be considered to optimize treatments and improve clinical outcomes. Furthermore, longitudinal studies have demonstrated variations in functional and anatomical responses to anti-VEGF therapy depending on patient baseline clinical characteristics (Tsilimbaris et al., 2016; Maguire et al., 2016). This underscores the potential value of an individualized approach, where each patient serves as their own control, to create more accurate and relevant predictive models. In this field, significant advancements have been made to develop robust statistical methodologies aimed at tracking individual patient evolution over time. In the realm of clinical metabolomics, the individualized approach to studying disease progression has garnered significant attention due to its potential to enhance diagnostic accuracy and treatment efficacy. Traditional models, which often rely on data aggregated from large cohorts, may overlook the unique metabolic profiles that characterize individual patients. This limitation can lead to misdiagnosis and ineffective treatment strategies, particularly in complex diseases where metabolic alterations are pivotal to understanding disease mechanisms and progression (Jacob et al., 2019; Palmnas and Vogel, 2013; Trivedi D et al., 2017).

Our results align precisely with this ideology. This study underscores the challenge of creating a global and comprehensive model using metabolomics to monitor and predict nAMD progression. These difficulties may stem from the initial experimental design, which, though well-aligned with clinical practice, produced incomplete and heterogeneous datasets. It also reflects the considerable diversity among patients themselves and the highly variable, often unpredictable disease progression. Our generated datasets showed limited variability across visits in both metabolite levels and ophthalmological changes related to the pathology. Yet, it is precisely these events and changes that should enable the construction of accurate models. Our analysis highlights the challenges of translating discoveries from highly controlled studies into real-world clinical practice, where numerous parameters, such as visit frequency, treatment adjustments, and disease progression, cannot be precisely controlled. These findings emphasize the importance but also the challenges of experimental design, particularly in longitudinal studies, and underscore the difficulties in monitoring and predicting patient progression over time and under treatment.

To develop predictive monitoring models, one solution would be to study a cohort of naïve patients who are followed from the initial detection of the pathology. Such patients generally adhere to standardized protocols in terms of visit frequency and injection schedules, providing a more consistent framework for analysis. An extended follow-up period of, for example, longer than 5 years would likely capture a sufficient number of significant events (such as transitions between active and inactive phases) to enable the assessment of potential links between nAMD progression, treatment responses, and metabolite levels. This cohort should account for the slow progression of the disease while also reflecting the realities of daily clinical practice to provide results that are both translatable and useful for clinicians.

## 5 Conclusion

This study raises the question of how metabolomics and its discoveries can be transferred from benchtop studies to clinical practices. Indeed, it highlights the complexity involved in designing, conducting, and valorizing metabolomics research when applied to slowly progressing and multifactorial diseases like nAMD.

At this stage, our study demonstrates several important points. First, it appears difficult to establish a global predictive model for disease progression or patient monitoring or to identify robust biomarkers that are common to all patients. Second, the construction of cohorts for longitudinal studies, and especially the selection of patients, remains challenging, as it is not easy to predict the future progression of patients, particularly with respect to treatment responses. Finally, our results reinforce the idea that this pathology must be approached in a more individualized manner, with the development of tools and models that allow for patient stratification and monitoring relative to their own baseline. Robust predictive models could be developed to monitor individual changes, flag abnormal trajectories, and predict patient evolution over time. We anticipated that, provided the appropriate experimental design was employed, robust predictive models could be developed, and some of our identified biomarkers could be useful for this development.

Future studies must account for the slow progression of the disease, the initial ophthalmological status of patients, and current patient management practices. They should also be multicentric to recruit a sufficient number of relevant and representative cases and use standardized protocols for recruitment, sample collection, and analysis. It is also essential, as we have done, to use appropriate and customized statistical tools for individualized assessments of disease progression, integrating metabolic changes and disease advancement with advanced data visualization. This study establishes a clear methodology that effectively identifies metabolites associated with clinical markers commonly used to assess patient status, even when working with a limited number of samples.

We are therefore confident that, considering both the challenges and difficulties identified in this work and the preliminary results presented here, it is possible to develop predictive and individualized models for monitoring patients with nAMD. Such models could greatly assist clinicians in providing better care for these patients.

## Data Availability

The raw data supporting the conclusions of this article will be made available by the authors, without undue reservation.
